# Anticipation of Personal Genomics Data Enhances Interest and Learning Environment in Genomics and Molecular Biology Undergraduate Courses

**DOI:** 10.1371/journal.pone.0133486

**Published:** 2015-08-04

**Authors:** K. Scott Weber, Jamie L. Jensen, Steven M. Johnson

**Affiliations:** 1 Department of Microbiology and Molecular Biology, Brigham Young University, Provo, Utah, United States of America; 2 Department of Biology, Brigham Young University, Provo, Utah, United States of America; Penn State College of Medicine, UNITED STATES

## Abstract

An important discussion at colleges is centered on determining more effective models for teaching undergraduates. As personalized genomics has become more common, we hypothesized it could be a valuable tool to make science education more hands on, personal, and engaging for college undergraduates. We hypothesized that providing students with personal genome testing kits would enhance the learning experience of students in two undergraduate courses at Brigham Young University: Advanced Molecular Biology and Genomics. These courses have an emphasis on personal genomics the last two weeks of the semester. Students taking these courses were given the option to receive personal genomics kits in 2014, whereas in 2015 they were not. Students sent their personal genomics samples in on their own and received the data after the course ended. We surveyed students in these courses before and after the two-week emphasis on personal genomics to collect data on whether anticipation of obtaining their own personal genomic data impacted undergraduate student learning. We also tested to see if specific personal genomic assignments improved the learning experience by analyzing the data from the undergraduate students who completed both the pre- and post-course surveys. Anticipation of personal genomic data significantly enhanced student interest and the learning environment based on the time students spent researching personal genomic material and their self-reported attitudes compared to those who did not anticipate getting their own data. Personal genomics homework assignments significantly enhanced the undergraduate student interest and learning based on the same criteria and a personal genomics quiz. We found that for the undergraduate students in both molecular biology and genomics courses, incorporation of personal genomic testing can be an effective educational tool in undergraduate science education.

## Introduction

Finding promising teaching practices for improving undergraduate science education is an important issue and evidence suggests a beneficial way to do this is to increase hands on experiential and active learning by working on real world issues and technologies [1–3]. Students today are coming of age in the genomics era and they will play an important role in shaping future ethical, medical, legal, and privacy issues related to how genetic information is viewed and used [4–7]. Personalized genomics is rapidly becoming more common in society and in the practice of medicine as costs fall and technologies improve [8–10]. While full genome sequencing remains cost prohibitive for most people, screening the genetic makeup of an individual by looking at over one million variations in small nucleotide polymorphisms (SNPs) is an inexpensive and developed alternative to determine the genetic makeup of an individual. Personal genomics companies offer direct-to-consumer tests that allow them to independently obtain genetic information about disease risk, drug sensitivities, traits, and ancestry [11–13]. The ethics of how this type of information should be used and how the government regulates this type of data is uncertain and provides a current and relevant classroom discussion topic [14–16]. Having access to this personalized genome technology provides a practical learning opportunity for students to not just discuss genetics and genomes in abstract, but to learn in a more active and relevant way as they analyze their own personal genomics data.

Researchers recently found that incorporating personal genome testing in the medical school classroom enhanced the learning experience of students on multiple levels. They concluded personal genome testing and using personal genotype data in the classroom enhanced both students’ self-reported and personal genomics based quiz scores [17]. We hypothesized that personal genomics data would be a valuable active-learning resource for improving undergraduate science education and hypothesized that the personalized nature of this type of information should provide students with increased interest in understanding genetics and its application. We specifically wanted to test if the anticipation of getting their data after the course ended could provide added interest and incentive for students to learn more. This hypothesis is based on motivational theory which would suggest that students will likely be intrinsically motivated by the possibility of receiving personal genomic data by responding to the value-related valence of interest, i.e., they find personal value in the task. And indeed, value-related tasks have been associated with deeper learning [18]. Other researchers have shown that using content personally relevant to a student will motivate them to participate more fully in the learning experience [19, 20].

We determine to test the hypothesis that anticipation of getting personal genome data increases student motivation in the subject and student learning by making the topic more relevant and personal. This was tested in two senior-level science courses at Brigham Young University [Advanced Molecular Biology (MMBIO 441) and Genomics (MMBIO 468)]. Of particular note, we found that student anticipation of obtaining their personal genomic data results in 1) increased self-reported motivation and learning based on survey responses, and 2) increased reported time working on personal genomics products.

## Materials and Methods

### Subjects

Subjects were undergraduate students enrolled in two senior-level undergraduate science courses at Brigham Young University [Advanced Molecular Biology (MMBIO 441) and Genomics (MMBIO 468)] offered in the Winter 2014 and 2015 semesters. The classes had approximately 40–50 students each and met three times per week for 50 minute time periods. All of the students enrolled in these two courses had previously completed an introductory molecular biology course (MMBIO 240). Students in the courses taught during the 2014 semester were given access to demo data sets and the option to receive personal genomics kits, whereas students in the 2015 semester were given access to demo data sets, but were not given kits to measure their own genomic data. Any students who had previously undergone personal genomics testing or did not complete both the pre and post surveys were not included in the data analysis. The personal genomics kits were purchased from 23andMe (https://www.23andme.com/academic/) at an academic price using funds from a Brigham Young University Teaching enhancement grant. In the winter semester of 2014 there were a total of 84 students who completed both the pre and post surveys and received personal genomics kits (42 students in Advanced Molecular Biology and 42 students in Genomics) and in the winter semester of 2015 there were 71 students who completed both the pre and post surveys and did not receive personal genomics kits (40 students Advanced Molecular Biology and 31 students in Genomics).

### Personal Genome Analysis

The Advanced Molecular Biology and Genomics courses both have a focus on personalized genomics for the last two weeks of the semester. During the semester, before starting the focus on genomics, students were asked to read articles to help them understand the pros and cons of personal genomics testing and students in the 2014 courses were given the option to decide whether or not they wanted a personal genome kit from 23andMe [21, 22]. The Brigham Young University Institutional Review Board approved the study methodology (Study #E14459). There was also a classroom discussion led by the instructors (K.S.W. and S.M.J.) reviewing the pros and cons. Students in the 2014 courses that decided to receive a kit collected their samples at home, sent them in to the company on their own, and received their data after the course was finished. This was done to safeguard student privacy, the kits were given to the students and they sent them into the company and were the only people with access to their genomic data.

As part of the two-week focus on personal genomics, the instructors (K.S.W. and S.M.J) described how the testing assays work and what can and cannot be concluded from this type of data. The instructors also demonstrated how demo genomic data could be accessed using an online demo data set provided by 23andMe. This demo data included genomic data for an anonymous family (Mendel family) as well as 12 diverse anonymous individuals. Students were given a packet with instructions on how to obtain access to these demo data sets as well as a step-by-step packet on analysis of Alzheimer’s disease. In the Advanced Molecular Biology course, completing this packet was optional for students, whereas in Genomics completion of this packet was coupled with a required homework assignment.

### Survey Instrument

At the start and conclusion of the two-week personal genomics emphasis, we administered a survey to measure the student attitudes and understanding of personal genomics and personalized medicine. These pre and post surveys were adapted from a survey used in the Stanford Medical Student study and assessed attitudes and knowledge about personal genomics testing and the classroom learning experience of the students during this two-week personal genomics emphasis in the courses [17]. Assessment of student attitudes was measured by yes/no questions or by agreement with statements on a 5-point Likert scale. We assessed student understanding of personal genomics by scoring a 10-question multiple-choice quiz. The responses and scores of students given the personal genomic kits (2014) were compared to students who were not given the kits (2015). Whether or not students were given personal genome kits, all students had access to the same demo genomic profiles of anonymous individuals. There were no minors or children included in this study. The survey contained a written consent form that was documented in each completed survey and the Brigham Young University Institutional Review Board approved the study methodology (Study #E14459).

### Data analysis

We analyzed responses from students who completed both the pre and post surveys and had not had their personal genome analysis done previously. Students who were enrolled in both courses and took the pre and post surveys had their responses counted only once. Student attitudes were assessed on a 5-point Likert scale and data comparisons between kit and no kit for Likert questions were performed using a Mann-Whitney U-test. Comparisons of student binary survey data and change in student hours were analyzed comparing kit and no kit responses using an independent-samples t-test. Comparisons of pre- and post-survey data were evaluated using a paired t-test. The difference in improvement between assignment and no-assignment students on a personal genomics quiz was assessed by an independent-samples t-test. All statistics were performed using Prism 6 software (GraphPad).

## Results

Eighty-four students completed both the pre and post surveys and received personal genomics kits (42 students in 2014 Advanced Molecular Biology course and 42 students in the 2014 Genomics course) and 71 students completed both the pre and post surveys and did not receive personal genomics kits (40 students in the 2015 Advanced Molecular Biology course and 31 students in the 2015 Genomics course). All of these students were undergraduates taking the Advanced Molecular Biology or Genomics courses at Brigham Young University during winter semester of 2014 or winter semester of 2015.

### Student attitudes about interpreting their own personal genome results

**Fig 1:**
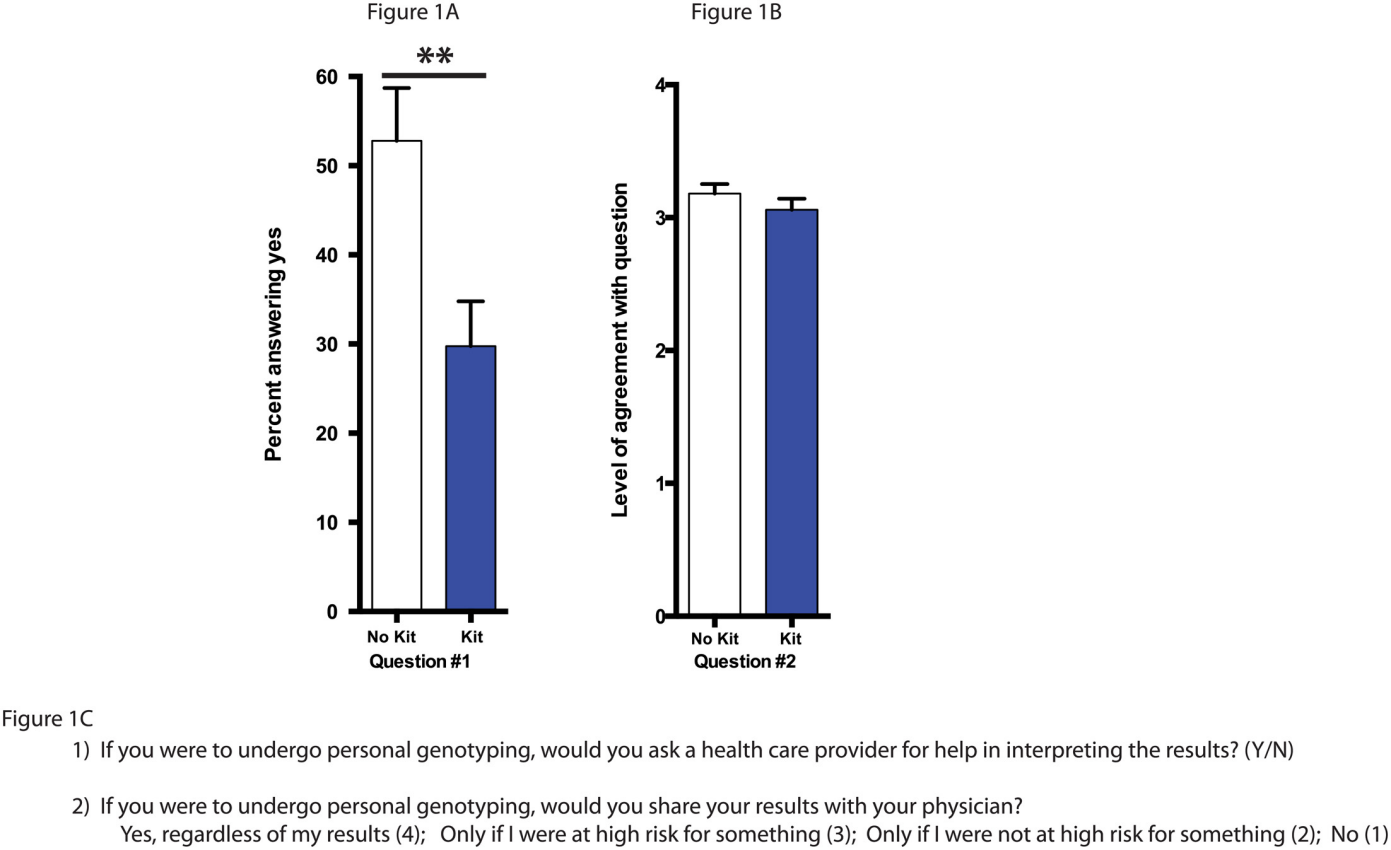
Student attitudes about sharing personal genomics data. A) Percentage of students answering “yes” to question #1 about whether they would ask health care workers for help in interpreting their results. B) Student responses to Question #2 regarding whether they would share their results with a physician. For A and B, statistical analysis performed using an unpaired Student t-test and all values are mean ± SEM with n = 71 for no kit (white), and n = 84 for kit (blue) (** = p<.01). C) Student survey questions 1–2.

As part of our study we surveyed student attitudes towards personal genome testing after our two-week focus on personal genomics. We asked students (Y/N) if they were to undergo personal genomics testing would they ask a health care provider for help in interpreting the results. We found that those who had submitted their personal genome kits and were anticipating receiving their data were significantly less likely to answer that they would ask a health care provider for help interpreting their results (Fig 1A and 1C; question 1; p<.01). There was no difference between groups as to whether or not they would share their results with a physician (Fig 1B and 1C; question 2). Students were given 4 options to answer this question and there is no dramatic difference in the percentage of students selecting each answer option (S1 Fig).

**Fig 2:**
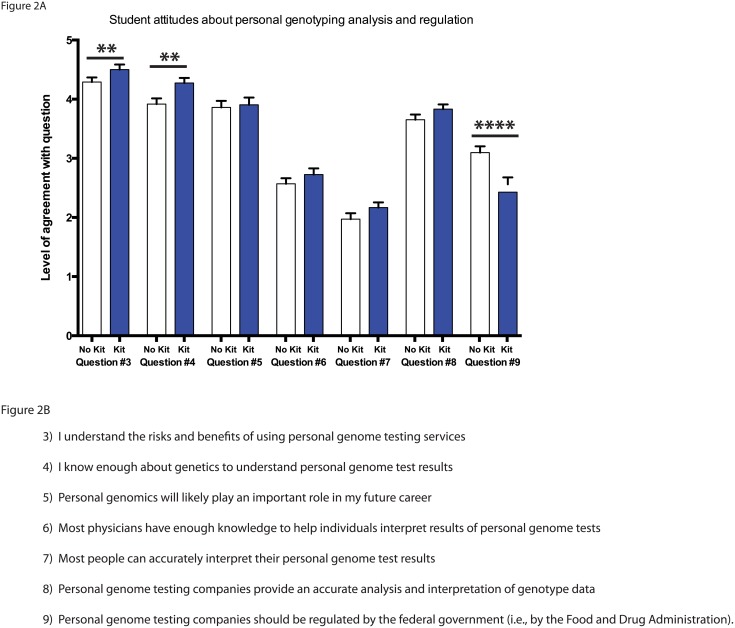
Student attitudes about personal genomics analysis and regulation. A) Student response to statements in questions 3–9 on a 5-point Likert scale. Statistical analysis performed using the Mann-Whitney U-test and all values are mean ± SEM with n = 71 for no kit (white), and n = 84 for kit (blue) (** = p<.01; **** = p<.0001). B) Student survey questions 3–9.

Students were asked to rate their confidence on a number of topics regarding their attitudes towards personal genotyping analysis and regulation using a 5-point Likert scale (Fig 2). Those students anticipating receiving their genomic data were significantly more confident in their understanding of the risks and benefits of using a personal-genome-testing service and in their ability to understand their personal genomics results (Fig 2A and 2B; questions 3 and 4; p<.01). Both groups had similar expectations for the role of personal genomics in their future career (Fig 2A and 2B; question 5).

### Student attitudes about others interpreting personal genome results

Students from both groups were asked about the ability of others to interpret personal genomics results and had similar levels of confidence in the abilities of physicians, other people, and personal genomic companies (Fig 2A and 2B; questions 6–8). There was a significantly different response between groups when they were asked if the federal government should regulate personal genomic testing companies. Those students who did not receive a personal genome kit were significantly more likely to agree that these companies should be regulated in comparison to the group that had submitted their genomes for analysis (Fig 2A and 2B; question 9; p<.0001).

### Personal genomics and classroom learning experience

**Fig 3:**
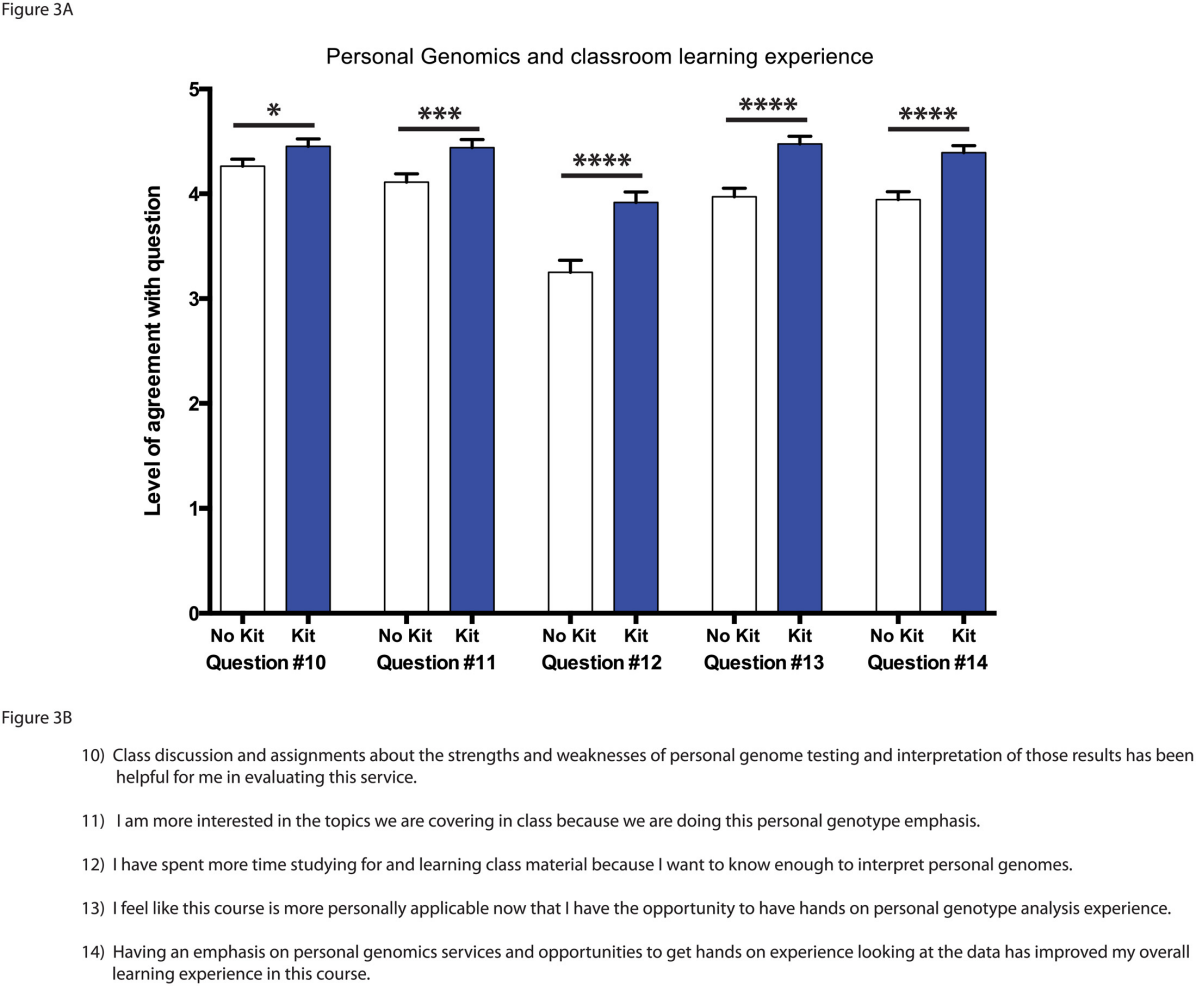
Student attitudes about personal genomics and the classroom learning experience. A) Student response to statements in questions 10–14 on a 5-point Likert scale. Statistical analysis performed using the Mann-Whitney U-test and all values are mean ± SEM with n = 71 for no kit (white), and n = 84 for kit (blue) (* = p<.05; *** = p<.001; **** = p<.0001). B) Student survey questions 10–14.

Students were also asked to rate their confidence on a number of topics regarding their attitudes towards personal genomics and their classroom learning experience using a 5-point Likert scale (Fig 3). Students who were expecting to analyze their personal genomic data were more confident in the value of the assignments and classroom discussions in helping them evaluate the personal genome services (Fig 3A and 3B; question 10; p<.05). Students who received the personal genomics kits and were anticipating analyzing their data had significantly higher agreement with the statement that they were more interested in the classroom topics because of the personal genomics emphasis (Fig 3A and 3B; question 11; p<.001). These same students said they spent significantly more time studying and learning class material because they wanted to know enough to interpret their personal genomics results (Fig 3A and 3B; question 12; p<.0001). They also had significantly higher agreement with the statement that the course was more personally applicable and that the personal genomics focus had improved their overall learning experience in the course (Fig 3A and 3B; questions 13 and 14; p<.0001).

### Evaluation of time spent using personal genomics products and personal genomics quiz scores

**Fig 4:**
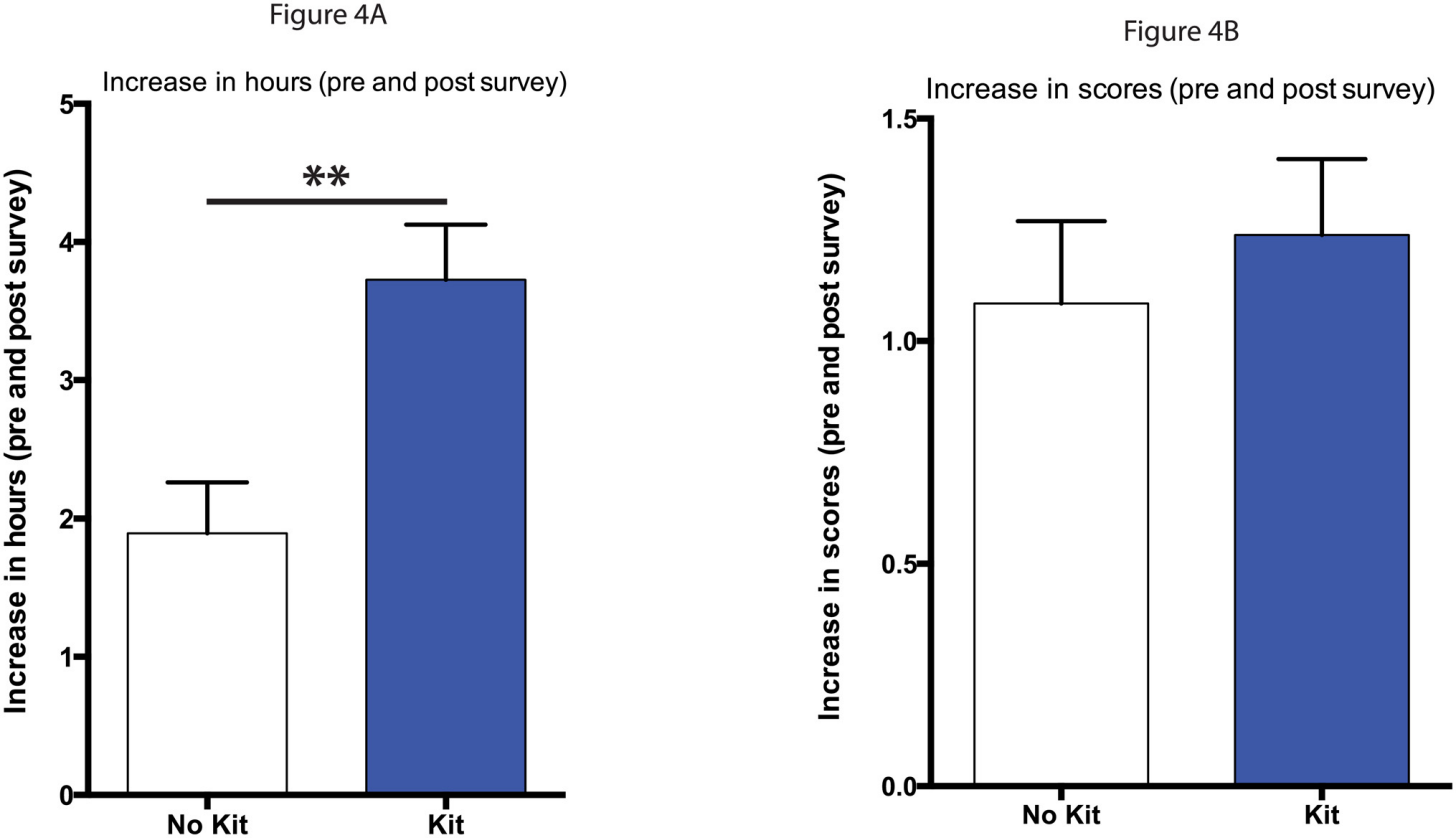
Change in hours spent using personal genomics products and scores on personal genomics quiz. A) Students reported hours spent using personal genomics products on pre and post surveys. The change in hours (increase) between the pre and post surveys were calculated and the data from students who did not receive kits (white) were compared with the data from students who did (blue). B) Student took a 10-question personal genomics quiz on pre- and post-surveys. The change in scores (increase) between the pre and post surveys were calculated and the data from students who did not receive kits (white) were compared with the data from students who did (blue). Statistical analysis for A and B were performed using an unpaired Student t-test and all values are mean ± SEM with n = 71 for no kit, and n = 84 for kit (** = p<.01).

In order to better quantify student interest in personal genomics, we asked students to report the amount of time they had spent using/researching personal genomics products before and after our two-week focus on personal genomics. Whether or not students were given personal genomics kits, they were given access to online personal genomics demo profiles. We found that both groups of students had spent significantly more time using these products by the end of the emphasis compared to the pre-survey hours (data not shown). We then calculated the change in hours (increase) between the pre and post surveys and compared the increase in hours of students who did not have the kit with those who did. When we did this we found that those that were given the kits spent significantly more time using personal genomics products, almost 2 hours more, than students who were not anticipating getting their own personal genomics data (Fig 4A; p<.01). We also evaluated student familiarity with personal genomics in these pre and post surveys with a 10-question personal genomics based quiz (S2 Fig). We found that student scores in both groups increased significantly in the post survey compared to the pre survey (data not shown). When we compared the increase in scores between the pre and post surveys of the groups there was not a significant difference in their improvement in scores whether or not they received a kit (Fig 4B).

### Role of genomic kits and homework on time spent using personal genomics products

**Fig 5:**
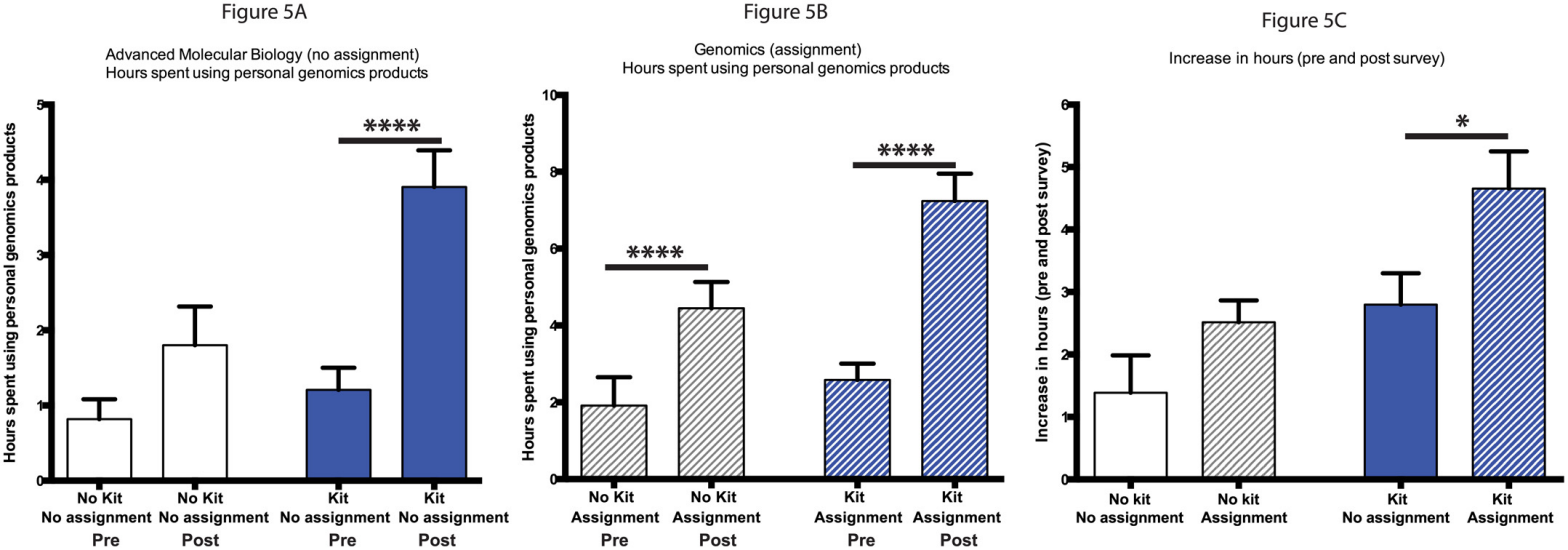
Effect of assignments and kits on time spent using personal genomics products. A) Reported hours spent using personal genomics products for students in Advanced Molecular Biology. Students in this class did not have a required homework assignment. The left two columns are the pre- and post-survey results from students who did not receive kits (white); whereas the right two columns are the survey results from students who did (blue). Statistical analysis performed using a paired t-test and all values are mean ± SEM with n = 40 for no kit/no assignment (white), and n = 42 for kit/no assignment (blue) (**** = p<.0001). B) Reported hours spent using personal genomics products for students in Genomics. These students had a required homework assignment. The left two columns are the pre- and post-survey results from students who did not receive kits (hatched white); whereas the right two columns are the survey results from students who did (hatched blue). Statistical analysis performed using a paired t-test and all values are mean ± SEM with n = 31 for no kit/assignment (hatched white), and n = 42 for kit/assignment (hatched blue) (**** = p<.0001). C) The change in hours (increase) between the pre and post tests for each course and group were calculated. In the left two columns were students who did not receive kits. Students who did not have a homework assignment (white) were compared to those that did (hatched white). In the right two columns all students received kits. Students that did not have a homework assignment (blue) were compared to those that did (hatched blue). Statistical analysis performed using an unpaired Student t-test and all values are mean ± SEM with n = 40 for no kit/no assignment (white), n = 42 for kit/no assignment (hatched white), n = 31 for no kit/assignment (blue), and n = 42 for kit/assignment (hatched blue) (* = p<.05).

As part of our study, we had one course (Genomics) that gave a specific personal-genomics assignment using the online, personal-genomics demo data for students to complete; whereas the other course (Advanced Molecular Biology) gave students an optional packet to guide them through how to access the online personal genomics demo profiles. This allowed us to evaluate the individual impact of kits and homework on the number of hours student spent researching personal genomics. When looking at the hours spent using personal genomics products, those that did not receive a kit or a graded assignment did not have significant differences in hours between pre and post surveys (Fig 5A; left two columns). Students who did not have an assignment, but received a kit, did have significant differences in hours between pre and post surveys (Fig 5A; right two columns; p<.0001). Those students who did not receive a kit, but had an assignment, had significant differences in hours between pre and post surveys (Fig 5B; left two columns; p<.0001). Students who received a kit and had an assignment spent even more hours using personal genomics products between pre and post surveys (Fig 5B; right two columns; p<.0001). It is important to note that the increase in hours spent using personal genomics products due to having a kit (without an assignment) was similar to the difference found when students have a kit with an assignment (Fig 5A and 5B; right two columns). This suggests that the increased time spent working on personal genomics is due to increased student interest and not due to problems completing an assignment or learning the material since even students who did not have an assignment to complete showed this increase.

We calculated the change in hours between the pre and post surveys (increase) for each of these groups and found that having an assignment did not significantly change the number of hours spent for those who did not receive a kit, but did significantly increase the hours spent by those that received a kit (Fig 5C; p<.05). It is interesting to note that the increase in hours due to an assignment (with no kit) was almost as big as the increase in hours due to having a kit without an assignment (Fig 5C, middle two columns). In terms of hours spent, it appears that the increase in time spent due to an assignment and the increase in time spent due to a kit are independent and additive (Fig 5C; right column).

### Role of genomic kits and homework on student personal genomics quiz scores

**Fig 6:**
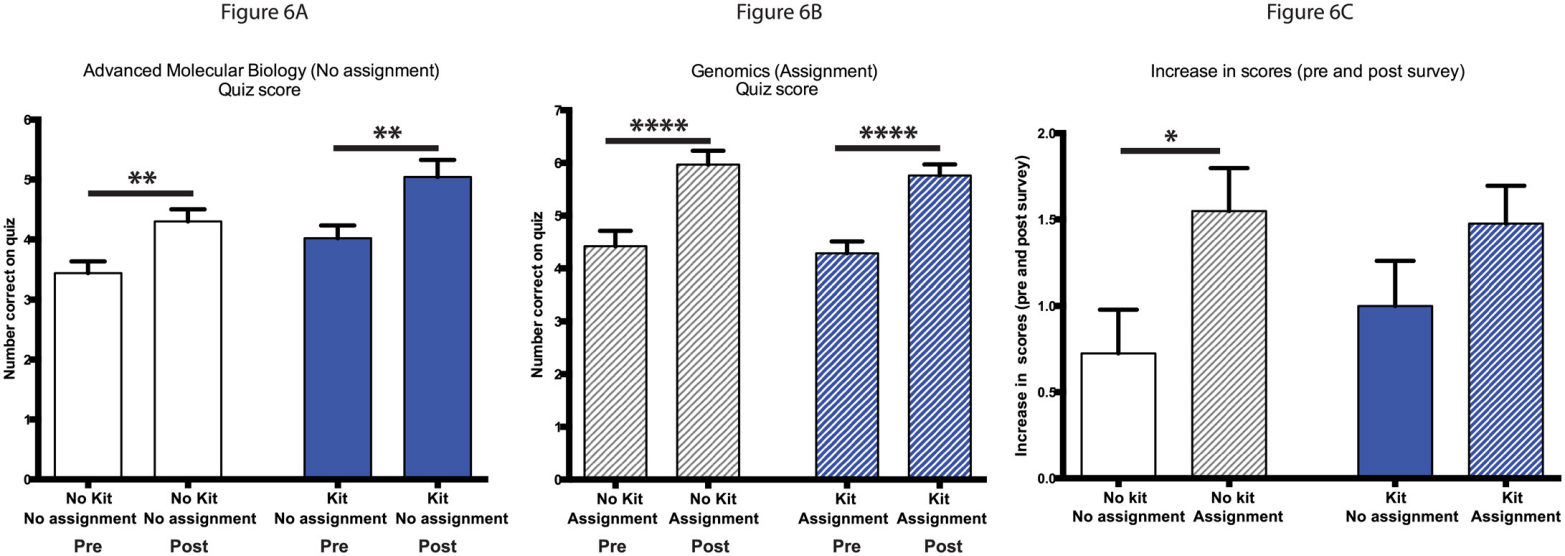
Effect of assignments and kits on student scores on 10-question personal genomics quiz. A) Scores on 10-question personal genomics quiz for students in Advanced Molecular Biology. Students in this class did not have a required homework assignment. The left two columns are the pre- and post-survey results from students who did not receive kits (white); whereas the right two columns are the survey results from students who did (blue). Statistical analysis performed using a paired t-test and all values are mean ± SEM with n = 40 for no kit/no assignment (white), and n = 42 for kit/no assignment (blue) (** = p<.01). B) Scores on 10-question personal genomics quiz for students in Genomics. These students had a required homework assignment. The left two columns are the pre- and post-survey results from students who did not receive kits (hatched white); whereas the right two columns are the survey results from students who did (hatched blue). Statistical analysis performed using a paired t-test and all values are mean ± SEM with n = 31 for no kit/assignment (hatched white), and n = 42 for kit/assignment (hatched blue) (**** = p<.0001). C) The change in scores (increase) between the pre and post tests for each course and group were calculated. In the left two columns were students who did not receive kits. Students who did not have a homework assignment (white) were compared to those that did (hatched white). In the right two columns, all students received kits. Students that did not have a homework assignment (blue) were compared to those that did (hatched blue). Statistical analysis performed using an unpaired Student t-test and all values are mean ± SEM with n = 40 for no kit/no assignment (white), n = 42 for kit/no assignment (hatched white), n = 31 for no kit/assignment (blue), and n = 42 for kit/assignment (hatched blue) (* = p<.05).

As mentioned above, the Genomics course had a required online personal-genomics homework assignment and the Advanced Molecular Biology had an optional packet outlining how to analyze online demo genomic data. This allowed us to evaluate the individual impact of kits and homework on student scores on the personal genomics quiz given during the pre and post surveys. We found that when students did not have the required assignment (with or without a kit), student scores significantly increased from the pre to post survey (Fig 6A; p<.01). When students were given a required assignment, scores for students increased significantly between pre and post surveys whether or not they received a personal genome kit (Fig 6B; p<.0001). We were then able to evaluate the change in scores between the pre and post surveys (increase) to further understand the role of assignments. When comparing the students who did not receive a kit, we found that students who were given the assignment had significantly higher increases in scores compared to those who were not given an assignment (Fig 6C; left two columns; p<.05). The same comparison between students who had received kits did not reveal significant differences between their increases in scores (Fig 6C). Comparison of the increase in scores for all students who received a kit to those students who did not received a kit revealed no significant differences (Fig 4B). Thus, student anticipation of obtaining their own personal genomics data did not result in improved personal genomic quiz scores, but did result in increased interest in both the self-reported survey and hours spent using personal genomics products as well as a self-reported improvement in the overall learning experience (Figs 3 and 4A).

## Discussion

We report here the first study, to our knowledge, to evaluate the benefits of incorporating personal genomics kits into an undergraduate science course and testing the effect of anticipation of obtaining personal genomic data on student interest and learning environment. Our results suggest that integration of personal genomics into undergraduate classrooms can be a valuable means of improving student interest and the learning environment when teaching the concepts of molecular biology and genomics. At the end of the course our students reported that anticipation of getting their personal genomics data had enhanced their learning experience, time spent learning about personal genomics, and overall attitude about the course in an undergraduate classroom.

In this study we tested the prediction that student anticipation of obtaining their personal genomics data enhances student interest and learning experience. Analysis of our data revealed that student anticipation of getting their own personal genomic data did increase student interest and the learning environment based on student reports and increased their reported time spent working on personal genomics. We also found anticipation of analyzing their own personal genomics data coupled with homework assignments resulted in even greater interest compared to students without homework.

While incorporating personalized genomics into the classroom can be a valuable educational tool, it also comes with a number of potential ethical issues that can be problematic. For example, the highest profile problem with personalized genomics and undergraduate education occurred in 2010 when U.C. Berkley started a “Bring your Genes to Cal” program for incoming freshman and transfer students that gave them the opportunity to submit their DNA for genetic analysis [23]. Initially the data was to be given directly to the students, but the California Department of Public health insisted that all DNA tests be ordered by a physician and analyzed by certified laboratories [23]. In response to this, U.C. Berkley decided to return only aggregate data to the students and the program was far less effective educationally and very controversial [24].

An important part of our study is that our students submitted their own samples and did not actually receive their personal genomic data until the course had ended. No one besides the student had access to his or her personal genomic data. While integrating analysis of personal genomics data as a part of the course could be a valuable way to enhance learning, it also produces additional classroom privacy issues that would need to be addressed [25]. Importantly in this study, we found that the mere anticipation of getting your personal genomics data after the course is over plays an important role in improving student interest and learning in undergraduate molecular biology and genomics classes at Brigham Young University. This study provides evidence that once students know that they will be given a personal genomics kit and can get their data after the course ends, they will put more time into learning the material and enjoy the course more. By providing the students the opportunity to obtain their own personal genomic analysis after the course has ended, and by using generic personal genomic data in the actual courses and homework, we effectively circumvented many of the ethical concerns discussed above without losing the benefit that comes from access to personal genomic data. Thus anticipation may be just as beneficial to student learning as actual access to their data. Indeed, this would follow from motivational theory. By providing personal genomic data, we are likely shifting student motivation from extrinsic (for the sake of a grade) to intrinsic (because they find personal relevance) [26]. This is evidenced by the equal increases in scores but significantly different increases in the hours spent on genomic tasks with or without homework when genomics kits were provided (Figs 5C and 6C; right two columns). Future studies would need to be designed to specifically test this hypothesis.

Upon completion of the two-week personal genomics focus, we found that students who were anticipating analyzing their data had spent more time using personal genomics products. This likely played a role in the increased confidence that these students had in their ability to interpret their personal genome results. We also found that these same students reported less support for health care providers having their data and less agreement that personal genome testing companies should be regulated. Our students also self reported being more interested in the class topics, spending more time studying and learning, feeling like the course was more applicable, and enjoying the learning experience more. We also examined the effects of homework with and without the students receiving the kit. A required homework assignment increased the hours spent using personal genomics products as well as learning based on scores on the personal genomics quiz (Figs 5C and 6C), suggesting potential beneficial additive effects in student interest in specific situations of coupling the kits with required homework.

This study has a large sample size and students from multiple classes, but does have several limitations when broadly generalizing these findings. All of this work was done at a single institution and it will be interesting to see other institutions examine student anticipation of personal genomics in enhancing learning and interest in molecular biology and genomics. These limitations notwithstanding, our study represents the first evidence that undergraduate student anticipation of receiving personal genomics data can enhance learning and interest. As personal genomics is more routinely incorporated into classroom education, additional randomized studies at multiple institutions can further enhance our understanding of what effect personal-genomics testing has upon undergraduate student learning.

We believe it is critical for undergraduate science educators to find practical and engaging ways to improve science education and personal genomics represents a current and powerful means of doing this. Personal genomics provides a valuable method of incorporating discussions and critical thought about rapidly evolving medical, ethical, and privacy issues that currently confront society and will help prepare our students to contribute to future policy discussions. Although further analysis of the value of using personal genomics in undergraduate education is necessary, we believe it is a powerful educational resource that should be thoughtfully incorporated into molecular biology and genomics education.

## Supporting Information

S1 Fig Percentage of students answering each option for question #2. Student responses to Question #2 regarding whether they would share their results with a physician. There were four answer options and percent response for each option is displayed. [Red = Yes, regardless of my results (4); Orange = Only if I were at high risk for something (3); Yellow = Only if I were not at high risk for something (2); and Green = No (1)].
(EPS)

S2 Fig 10-question genetics quiz. (PDF)
