# Supplementary material for: Anticipation of Personal Genomics Data Enhances Interest and Learning Environment in Genomics and Molecular Biology Undergraduate Courses
Source: PLoS One. 2015 Aug 4;10(8):e0133486. doi: 10.1371/journal.pone.0133486 (PMC4524698; doi:10.1371/journal.pone.0133486)
Supplement: S2 Fig — (PDF) [file pone.0133486.s002.pdf]

Supplemental Figure #2 – Personal Genomics Quiz

Knowledge of personal genomics

Please select the most appropriate answer choice.

1. The personal genome testing done by 23andMe uses which of the following to determine your genetic makeup:

    A. Full genome sequencing
    B. Whole exome sequencing
    C. Single nucleotide polymorphisms
    D. ChipSeq analysis
    E. RNA sequencing

2. What is the estimated heritability of Alzheimer’s Disease?

    A. 100%
    B. 60-80%
    C. 25-33%
    D. 5-10%
    E. 0%

3. What is the APOE gene?

    A. Encodes for Another Protein Overexpressed in the Elderly
    B. Critical for cervical balance and relay centers
    C. Apolipoprotein E, a cholesterol carrier found in the brain
    D. A dominant negative recessive mutant
    E. The driving factor for mad cow disease

4. What are the variants of APOE?

    A. ε2, ε3, ε4
    B. short, twisted, and frazzled
    C. β1, β2, β3, β4,
    D. Sonic hedgehog smooth and rough
    E. r1245 and rs356

5. How many genetic loci are used by 23andMe to determine the APOE variants?

    A. 1
    B. 2
    C. 3
    D. 4
    E. 5

6. The odds calculator takes into account all of the following <u>except</u>:

A. Ethnicity of individual
B. Age of individual
C. Sex of individual
D. Lifestyle of individual
E. Disease free status of individual

7. Which of the following genes is most commonly associated with autoimmune diseases?

A. Cox2 inhibitors intolerance
B. Major histocompatablity complexes
C. T cell receptors specific for hemoglobin
D. B cell deficiencies
E. Insulin trafficking

8. Which of the following contributes the most to an individuals breast cancer risk?

A. BRCA1
B. BRCA2
C. Environmental factors
D. FGFR2
E. CASP8

9. Why would a strong family history of breast cancer suggest you should get additional tests even if your 23andMe results are negative?

A. You don't need to get additional tests because you are fine
B. Effects of rare mutations not tested outweigh the risks of those reported
C. Getting a mammogram is the only reliable predictor of disease
D. Genes are not important in risk for breast cancer
E. You might get additional mutations after you take the test

10. Which of the following genes can be tested to determine resistance to HIV infection?

A. OCA2
B. BRCA1
C. CCR5Δ32
D. ACTN3
E. BFDRΔ7

Key – 1(c), 2(b), 3(c), 4(a), 5(b), 6(d), 7(b), 8(c), 9(b), 10(c)
